# Persistence of functional sympatholysis post-exercise in human skeletal muscle

**DOI:** 10.3389/fphys.2013.00131

**Published:** 2013-06-11

**Authors:** Jaclyn Moynes, Robert F. Bentley, Michael Bravo, J. Mikhail Kellawan, Michael E. Tschakovsky

**Affiliations:** Human Vascular Control Laboratory, School of Kinesiology and Health Studies, Queen's UniversityKingston, ON, Canada

**Keywords:** functional sympatholysis, skeletal muscle blood flow, sympathetic vasoconstriction, exercise, cold pressor test

## Abstract

Blunting of sympathetic vasoconstriction in exercising muscle is well-established. Whether it persists during the early post-exercise period is unknown. This study tested the hypothesis that it persists in human skeletal muscle during the first 10 min of recovery from exercise. Eight healthy young males (21.4 ± 0.8 yrs, SE) performed 7 min of forearm rhythmic isometric handgrip exercise at 15% below forearm critical force (fCF). In separate trials, a cold pressor test (CPT) of 2 min duration was used to evoke forearm sympathetic vasoconstriction in each of Rest (R), Steady State Exercise (Ex), 2–4 min Post-Exercise (PE_early_), and 8–10 min Post-Exercise (PE_late_). A 7 min control exercise trial with no CPT was also performed. Exercising forearm brachial artery blood flow, arterial blood pressure, cardiac output (CO), heart rate (HR), forearm deep venous catecholamine concentration, and arterialized venous catecholamine concentration were obtained immediately prior to and following the CPT in each trial. CPT resulted in a significant increase in forearm venous plasma norepinephrine concentration in all trials (*P* = 0.007), but no change in arterialized plasma norepinephrine (*P* = 0.32). CPT did not change forearm venous plasma epinephrine (*P* = 0.596) or arterialized plasma epinephrine concentration (*P* = 0.15). As assessed by the %reduction in forearm vascular conductance (FVC) the CPT evoked a robust vasoconstriction at rest that was severely blunted in exercise (R = −39.9 ± 4.6% vs. Ex = 5.5 ± 7.4%, *P* < 0.001). This blunting of vasoconstriction persisted at PE_early_ (-12.3 ± 10.1%, *P* = 0.02) and PE_late_ (-18.1 ± 8.2%, *P* = 0.03) post-exercise. In conclusion, functional sympatholysis remains evident in human skeletal muscle as much as 10 min after the end of a bout of forearm exercise. Persistence of functional sympatholysis may have important implications for blood pressure regulation in the face of a challenge to blood pressure following exercise.

## Introduction

During skeletal muscle exercise, increases in exercising skeletal muscle vascular conductance paired with elevations in cardiac output (CO) act to increase muscle oxygen delivery in order to meet metabolic demand (Andersen and Saltin, 1985; Koskolou et al., 1997). Although exercising skeletal muscle vascular conductance increases, the magnitude of this increase appears to be restrained by increases in sympathetic vasoconstrictor influence, regardless of whether the exercise involves a small muscle mass (Joyner et al., 1992) or a large muscle mass (Rowell, 1997). Both baroreflex (Keller et al., 2004) and metaboreflex-mediated (Seals and Victor, 1991) increases in muscle sympathetic nerve activity (MSNA) can contribute to this sympathetic restraint. In the case of large muscle mass exercise at higher intensities, this restraint is believed to be essential for preventing skeletal muscle vasodilation from exceeding CO and threatening arterial blood pressure during exercise (Rowell, 1997; O'Leary et al., 1997).

While MSNA in exercising muscle maintains its effectiveness as part of blood pressure regulation (Keller et al., 2004), the responsiveness of resistance vessels to it is blunted. This blunting was first identified by Remensnyder et al. (1962) in an *in situ* animal model and termed functional sympatholysis. Over the past ~15 years, it has been clearly established that functional sympatholysis occurs in exercising human skeletal muscle of both the arm and leg (Hansen et al., 2000; Tschakovsky et al., 2002; Dinenno et al., 2005; Watanabe et al., 2007; Fadel et al., 2012; Mortensen et al., 2012; Saltin and Mortensen, 2012). Its magnitude increases with exercise intensity, at least in the forearm (Tschakovsky et al., 2002; Watanabe et al., 2007).

Whether such blunting of sympathetic vasoconstriction persists following exercise, and if so for how long, has received less attention. DiCarlo's group has explored this in rabbit and rodent exercise studies and identified that between 10 and 26 min post-exercise blunted vasoconstriction in response to the α_1_-receptor agonist phenylephrine remained in evidence (Howard and DiCarlo, 1992; Patil et al., 1993). In humans, Halliwill and colleagues have investigated the role of sympathetic vasomotor control in explaining the post-exercise hypotension and increased resting vasodilation in the exercised muscles that persists for a number of hours following cycling exercise (Halliwill et al., 1996, 2003). Their first study identified a decrease in MSNA at rest and in response to a hypotensive challenge (sodium nitroprusside bolus infusion) ~60 min post-exercise, as well as a reduced calf vasoconstrictor response per unit MSNA increase evoked by ischemic forearm exercise ~90 min post-exercise (Halliwill et al., 1996). Their second study identified intact sympathetic vasoconstriction in the exercised legs in response to infusion of selective α_1_ and α_2_ agonists ~60 min following cycling exercise (Halliwill et al., 2003).

To our knowledge, these are the only studies to date which have investigated impaired post-exercise sympathetic vasoconstriction in humans. The current interpretation of their findings is that in humans the impaired transduction of MSNA to vasoconstriction an hour or more after exercise is due to pre-junctional not post-junctional (α receptor response) mechanism(s). Since functional sympatholysis during exercise has repeatedly been demonstrated to be a post-junctional phenomenon in humans (Rosenmeier et al., 2003; Dinenno et al., 2005; Kirby et al., 2008) blunted transduction of MSNA to vasoconstriction so long after exercise cessation may represent a delayed alternate “sympatholytic” mechanism.

To date, blunting of sympathetic vasoconstriction during the very early post-exercise recovery period (0–10 min) has not been investigated in humans. This is likely because quantification of sympathetic vasoconstriction during the non-steady state of exercise recovery is problematic. Potential mechanisms of functional sympatholysis include NO and Prostaglandins (Thomas and Victor, 1998; Chavoshan et al., 2002; Dinenno and Joyner, 2004), ATP (Rosenmeier et al., 2004; Kirby et al., 2008) and most recently metabolic acidosis (Ives et al., 2012). Of these, only NO's role in elevated skeletal muscle blood flow immediately post-exercise (Shoemaker et al., 1997; Radegran and Saltin, 1999) has been explored, with findings suggesting that it is a contributor. However, elevated plasma [ATP] during (Gonzalez-Alonso et al., 2002) and acutely following (Yegutkin et al., 2007) exercise, as well as a sustained reduction in venous effluent pH for a number of minutes following intense exercise have also been observed (Wiltshire et al., 2010). This would suggest that persistence of functional sympatholysis during the immediate post-exercise period is plausible in humans. The inevitable decline in sympatholytic molecules might dictate the time course of diminishing functional sympatholytic potency.

Therefore, we developed and validated a curve-fitting approach to allow quantification of sympathetic vasoconstriction in the immediate post-exercise non-steady state. We used this to test the hypothesis that functional sympatholysis persists in human skeletal muscle during the first 10 min of recovery from exercise.

## Materials and methods

### General methods

#### Subjects

Originally, 10 healthy males [*age* = 21.4 ± 0.8 years (mean ± SE)] volunteered to participate in this study. Data from two had to be omitted from final analysis due to issues discussed later. Therefore, data analysis and interpretation was based on an *n* = 8. They were non-obese, non-smokers, normotensive, and had similar physical activity levels as they commonly engaged in moderate bouts of walking, intramural sports, and occasional full body workouts (Table 2). This experiment was approved by the Queen's University Health Sciences and Affiliated Teaching Hospitals Research Ethics Board, in compliance with the terms of the Declaration of Helsinki. After receiving a complete verbal and written description of the experimental protocol and associated potential risks, each subject provided written, informed consent to participate in the outlined experiment.

#### Maximum voluntary contraction (MVC)

Maximum voluntary contraction (MVC) strength for each subject was determined as the peak force in kg of three 1–2 s maximal isometric handgrip contractions while laying supine with the arm extended to the side at heart level. Each contraction was followed by 1 min of recovery prior to the next contraction. Subjects were instructed to recruit only the forearm muscles during the voluntary contraction, so that the MVC performed would be representative of forearm handgrip strength in the position that was used for the experimental exercise protocol.

#### Forearm critical force (fCF) exercise test

Each subject underwent a single bout forearm critical force (fCF) exercise test in order to determine the work rate they would exercise at during the forearm exercise protocol. The fCF exercise task consisted of 10 min of rhythmic isometric maximal forearm handgrip contractions at a duty cycle of 1:2 s (1 s contraction, 2 s relaxation) with the handgrip dynamometer. The force output from the dynamometer and the contraction duty cycle were displayed to the subject throughout the exercise test. The subject was instructed to attempt to reach their MVC force output on each contraction over the 10 min. Each participant's fCF was calculated as the average power output of the last 30 s of the exercise test (Kellawan et al., 2010).

The fCF is the highest exercise intensity at which a metabolic steady state (plateau of VO_2_ and high energy phosphates) still occurs (Poole et al., 1988; Jones et al., 2008). As such, it represents a “functional peak aerobic power,” as it is the highest exercise intensity that can be maintained via aerobic ATP production without requiring supplemental ATP production via substrate level phosphorylation. It is commonly described as the boundary between heavy and severe exercise (Vanhatalo et al., 2007).

#### Forearm exercise at 15% below forearm critical force

Forearm exercise designed to evoke forearm functional sympatholysis consisted of 7 min of rhythmic isometric forearm handgrip contractions at a duty cycle of 1.2 s at 15% below the subject's fCF. This exercise intensity was chosen to maximize the potential for functional sympatholysis since it is exercise intensity-dependent (Tschakovsky et al., 2002), while still achieving a steady state exercise response (Moritani et al., 1981; Jones et al., 2008). As the active muscle mass is small, a central cardiovascular challenge was not expected while investigating the local vascular phenomenon of functional sympatholysis. The target force of 15% below fCF was displayed on the feedback monitor during the task. The exercise task was followed by 15 min of supine recovery.

#### Cold pressor test (CPT)

In this study, activation of sympathetic vasoconstriction with the subject laying supine was evoked using a CPT. The CPT required the subject to place a foot into a prepared ice bath (0–3°C) for 2 min. Subjects were instructed to rest their foot at the bottom of the ice bath, remain relaxed, avoid tensing their muscles, and concentrate on their breathing to maintain a normal breathing pattern rather than holding their breath at any time during the CPT.

#### Arterialized blood sampling for catecholamines

Subjects had their right hand heated with a standard 50 W heating pad (Sunbeam, Jarden Consumer Solutions, Canada) for ~10 min. Following hand heating, a 1-mL venous blood sample was taken from a dorsal hand vein or superficial vein of the wrist with an appropriately positioned butterfly needle (retrograde) or 20-gauge intravenous catheter (antegrade). The use of venous blood samples obtained from a heated vein has been previously demonstrated to accurately reflect the arterial concentration of lactate (Zavorsky et al., 2005). Thus, it was expected that other arterial plasma constituents, such as catecholamines, would also be representative.

The collected blood sample was immediately analyzed (StatProfile M Blood Gas Analyzer; Nova Biomedical, Mississauga, ON) to determine the hemoglobin oxygen saturation (%SO_2_). Adequate arterialization was defined as greater than 90% SO_2_. If appropriate arterialization was not achieved, further hand heating and subsequent blood sampling was required until a sample with SO_2_ of greater than 90% was obtained. When this was confirmed, a blood sample of 3 mL was initially drawn and discarded, followed by two 6 mL blood samples collected into EDTA vacutainer tubes.

#### Forearm deep venous blood sampling for catecholamines

A 20-guage catheter was inserted retrograde into an antecubital vein of the participant's right arm (non-experimental arm). Echo ultrasound was utilized prior to catheterization in order to ensure the selected vein drained forearm muscle (cephalic or median cubital vein) and was not a superficial vein.

Venous blood samples were drawn immediately pre- and post-CPT in order to assess the forearm catecholamine response to the CPT. Samples were withdrawn from the non-exercising arm as the sympathetic neural activation due to the CPT would be the same for both arms, and the right arm would not have the confound of different blood flows between rest, exercise and recovery. A blood sample of 3 mL was initially drawn and discarded, followed by two 6 mL blood samples collected into EDTA vacutainer tubes at each sample period.

#### Central hemodynamic monitoring

Heart rate (HR) was measured beat-by-beat with an electrocardiogram 3-lead placement. Arterial blood pressure for the determination of mean arterial pressure (MAP) was measured continuously throughout the study via finger photoplethysmography (Finometer MIDI, Finapres Medical Systems, Amsterdam, Netherlands) on a finger of the non-exercising hand. Stroke volume (SV) was estimated from the blood pressure waveform (Beatscope Easy ModelFlow™ method, Finapres Medical Systems, Amsterdam, Netherlands) and the same software calculated CO as HR × SV.

#### Brachial artery diameter and mean blood velocity (MBV)

For the left arm which was used to perform the exercise (experimental arm), the brachial artery was imaged through the use of a linear 10 MHz echo ultrasound probe, operating in two-dimensional B-mode (Vingmed System FiVe; GE Medical Systems, London, ON). MBV was measured with a 4 MHz pulsed Doppler ultrasound probe (Model 500V TCD; Multigon Industries, Mt. Vernon, NY), which was secured to the skin at a location ~5–10 cm distal to the Echo probe and proximal to the antecubital fossa. This position was marked on the skin to ensure the same probe position for experimental trials occurring on separate days. MBV was measured and recorded continuously on a personal computer data acquisition system. The probe insonation angle when parallel to the artery was 57°. The insonation angle was corrected based on imaging the vessel orientation relative to the skin surface at the Pulsed Doppler probe site. This correction adjusts the mean blood velocity voltage output that was calibrated at the probe insonation angle. For details see Pyke et al. (2008).

### Subject screening and familiarization

Subjects were instructed to abstain from consuming alcohol and/or caffeine 12 h prior and any food 4 h prior to the screening trial. Each subject participated in an fCF test, and practiced the constant work rate forearm exercise to become familiar with the exercise and be able to perform consistent contractions. In addition, each subject participated in the following screening activities.

#### 7-day physical activity recall questionnaire (7-PAR)

Each subject completed a 7-PAR during the initial screening and familiarization visit to the laboratory. The 7-PAR was used to characterize their average level of physical activity prior to completing the study. The use of the 7-PAR has previously been demonstrated as a reliable and valid method of quantifying physical activity (Taylor et al., 1984; Dishman and Steinhardt, 1988).

#### Echo and doppler ultrasound screening

Initial echo and pulsed Doppler ultrasound screening of the subject's experimental arm was necessary in order to ensure that a clear artery image and a blood velocity signal which did not contain signal from veins adjacent to the brachial artery could be obtained at rest, during and after forearm exercise. Subjects were instructed to lay supine on the laboratory bed while the investigator obtained a brachial artery image and brachial artery blood velocity signal as previously described. The subject then performed submaximal intensity rhythmic forearm handgrip contractions for a few minutes during which signal quality was assessed. No prospective subjects were turned away due to a poor brachial artery image or blood velocity signal.

#### CPT screening

Since the assessment of functional sympatholysis requires an experimental manipulation that elevates sympathetic vasoconstriction, it was necessary to select subjects in which a sympathetic vasoconstrictor response to the CPT could be confirmed. Therefore, all interested subjects completed CPT screening in order to confirm that the pressor response to the CPT was primarily due to increased TPR (which indicates sympathetic vasoconstrictor activation) rather than elevations of CO. The outlined CPT screening protocol was repeated approximately 3–4 months following the initial screening visit in order to determine the repeatability of each participant's response to the CPT. Of the 27 interested in participating, 24 returned to the laboratory for a follow-up screening. Of these 24 individuals, 10 demonstrated a clear, consistent TPR response to the CPT. These TPR responders were the final subjects selected to participate in the experiment. Of these 10, two were not included in data analysis; one due to poor quality Doppler velocity signal and the other due to lack of vasoconstrictor response to CPT. Therefore, an *n* = 8 was used for data analysis.

### Experimental protocols

**Figure 1:**
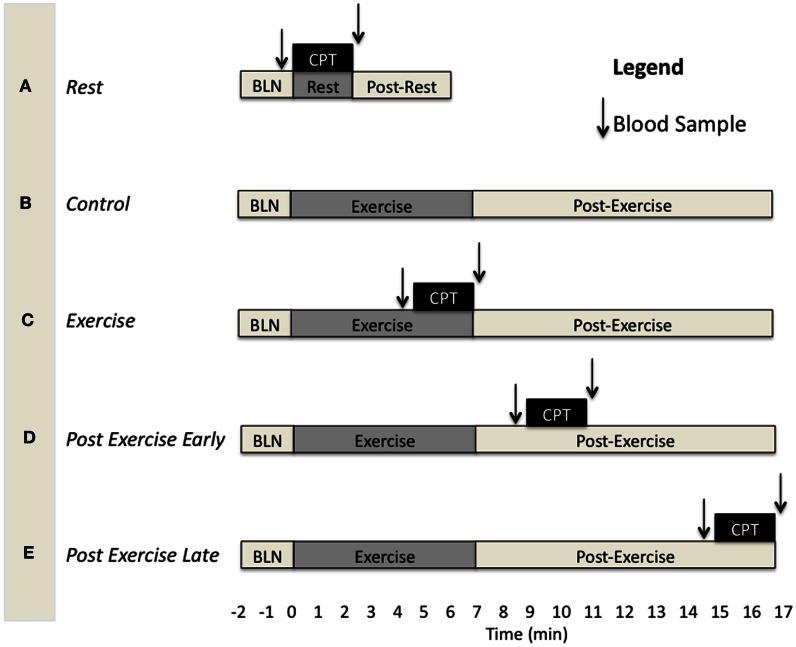
**Schematic depicting the different experimental trials and the timing of blood samples and the CPT for each**. BLN, resting baseline. CPT, 2 min Cold pressor test. **(A)** CPT during rest. **(B)** Exercise trial without CPT. **(C)** CPT during exercise. **(D)** CPT at 2–4 min of the post-exercise period. **(E)** CPT at 8–10 min of the post-exercise period.

Subjects came to the laboratory on three separate days for three separate experimental protocols having abstained from consuming alcohol and/or caffeine for at least 12 h and food for at least 4 h. They were instructed to lie supine on the laboratory bed with both arms supported to the side at heart level and instrumented for data collection while resting quietly. The experimental protocols were designed to assess functional sympatholysis during exercise (Ex), at 4 min post-exercise (PE_early_), and at 10 min post-exercise (PE_late_). Each of these protocols consisted of three different experimental trials (Figure 1). The first trial was always a resting CPT trial (**A** in Figure 1) which consisted of 2 min resting baseline, followed by 2 min of the CPT, and finally another 2 min post-CPT. The second trial was either a control exercise trial (**B** in Figure 1), consisting of 2 min resting baseline, followed by 7 min forearm exercise at 15% below fCF, and finally 10 min of post-exercise recovery, or a CPT trial (one of either exercise CPT, PE_early_ CPT or PE_late_ CPT (**C–E** in Figure 1). The order in which a subject performed trial B vs. either of C, D, or E was consistent across protocols within subjects, but counter-balanced between subjects. For example, one subject may have performed trials on the three different days in the order A, B, C and therefore the other days would be A, B, E and A, B, D. Another may have performed them in order A, C, B and therefore the other days would be A, D, B and A, E, B. Each experimental trial was separated by at least 10 min of recovery in order to allow variables to return to baseline values. As there were two CPT's within each experimental protocol, the subject alternated the foot that was placed in the ice bath.

In order to confirm that the vasoconstrictor response to a second CPT was not blunted, we performed additional time control experiments (*n* = 7) in which a CPT was administered twice under resting conditions in one laboratory visit. The time between the two CPT tests was equivalent to the time interval that would have occurred between the rest CPT and half way between the time of the PE_early_ and PE_late_ CPT's.

### Data acquisition and analysis

#### Central hemodynamic response to CPT

For all trials shown in Figure 1, central hemodynamic measurements were recorded continuously for the duration of each trial at 200 Hz using a Powerlab™ data acquisition system (ADInstruments, Sydney, Australia). To quantify the effect of the CPT on central hemodynamics and confirm that it was consistent across days, beat-by-beat measurements of mean arterial blood pressure (MAP), and calculated CO and TPR (MAP/CO) during the rest trial on the PE_early_ and PE_late_ days were averaged over 30 s at the following time points: baseline immediately prior to start of CPT, and 30, 60, 90, and 120 s of the CPT.

#### Forearm hemodynamic response to CPT

***Brachial artery mean blood velocity (MBV).*** Brachial artery MBV was recorded continuously for the duration of each trial at 200 Hz using a Powerlab™ data acquisition system (ADInstruments, Sydney, Australia). Brachial artery MBV for the purpose of assessing the forearm hemodynamic response to CPT at rest and during exercise was the average MBV over the 30 s period immediately prior to the start of the CPT and the last 30 s of the CPT. For the post-exercise period the continuous measures of MBV were used in conjunction with brachial artery diameter (see below) to obtain a continuous FBF profile post-exercise.

***Brachial artery diameter (BAD).*** Brachial artery images were recorded continuously in Digital Imaging and Communications in Medicine (DICOM) format and stored on a personal computer for offline analysis. BAD was analyzed via custom built automated edge-detection software (Woodman et al., 2001), which allows the user to identify a region of interest where the vessel walls are the clearest. The arterial walls are then tracked by detecting the greatest change in pixel brightness in the vertical axis. The edge-detection software collects one diameter measurement for every pixel column and uses the median diameter as the diameter for that frame.

With this approach, brachial artery diameter measurements for the purpose of assessing the forearm hemodynamic response to CPT at rest and during exercise were obtained at 5 s increments over the 30 s period immediately prior to the start of the CPT and the last 30 s of the CPT. These 5 s increment measurements were averaged to obtain a single diameter measurement that was used with the corresponding MBV to calculate FBF for rest and exercise pre- and end-CPT, respectively. For post-exercise, diameter measurements were obtained every 20 s. These were then curve fit (mono-exponential decay) with statistical software (SigmaPlot 11.0, Systat Software, Inc.) to provide a continuous diameter estimate and allow forearm blood flow calculations for the entire recovery period.

***Forearm blood flow (FBF).*** FBF (ml/min) was calculated as follows: FBF = [MBV × 60 × π × (BAD/2)^2^], where MBV is cm/s, 60 is s/min, BAD is cm.

In the case of the Rest and Exercise CPT experimental trials, where there is a steady state in the absence of CPT, FBF was derived from the 30 s averaged MBV and brachial artery diameter at the pre- and end-CPT time points. In this way, CPT-evoked sympathetic vasoconstriction is derived by comparing pre- and end-CPT time points. In the case of the Post-Exercise CPT experimental trials, the curve fit of the diameter measurements provided beat-by-beat diameter values corresponding to the beat-by-beat MBV, allowing beat-by-beat FBF calculation (see below for explanation of analysis required for quantifying CPT-evoked sympathetic vasoconstriction during the non-steady state post-exercise period).

***Forearm vascular conductance (FVC).*** FVC (ml/min/100 mmHg) was calculated as follows: FVC = FBF/MAP × 100, where MAP is mmHg and multiplying by 100 just allows FVC values to be in the same order of magnitude as FBF.

***Sympatholysis during rest and exercise.*** In the case of the Rest and Exercise CPT experimental trials, where there is a steady state in the absence of CPT, FVC was derived from the 30 s averaged FBF and MAP at the pre- and end-CPT time points. The vasoconstrictor effect of the CPT in the experimental forearm in these trials was calculated as the %reduction in FVC = [(FVC_post−CPT_ − FVC_pre−CPT_)/FVC_pre−CPT_] × 100, where FVC_post−CPT_ is the FVC averaged over the last 30 s of the CPT and FVC_pre−CPT_ is the FVC averaged over the 30 s immediately prior to the CPT. A blunting of the %reduction in FVC in response to the CPT during exercise compared to rest was interpreted as representing functional sympatholysis (Tschakovsky et al., 2002).

***Sympatholysis during post-exercise recovery.*** In the case of the Post-Exercise CPT experimental trials, beat-by-beat FBF obtained as described previously, was combined with beat-by-beat MAP to obtain beat-by-beat FVC = FBF/MAP × 100 for the entire recovery period. There is no steady-state FBF, FVC, or MAP in the absence of CPT during the post-exercise recovery phase since these are changing over time as a function of normal recovery from exercise. Therefore, one cannot use a pre- vs. end-CPT comparison to quantify the CPT vasoconstrictor effect. To overcome this, we developed an analysis approach that allowed us to determine what the FVC would have been during the last 30 s of the CPT if the CPT had not occurred (i.e., FVC_predicted_; what the normal recovery FVC at that time would have been). The difference between this and the actual FVC (FVC_actual_) in the last 30 s of the CPT would therefore constitute the degree of vasoconstriction evoked by the CPT during recovery from exercise.

**Figure 2:**
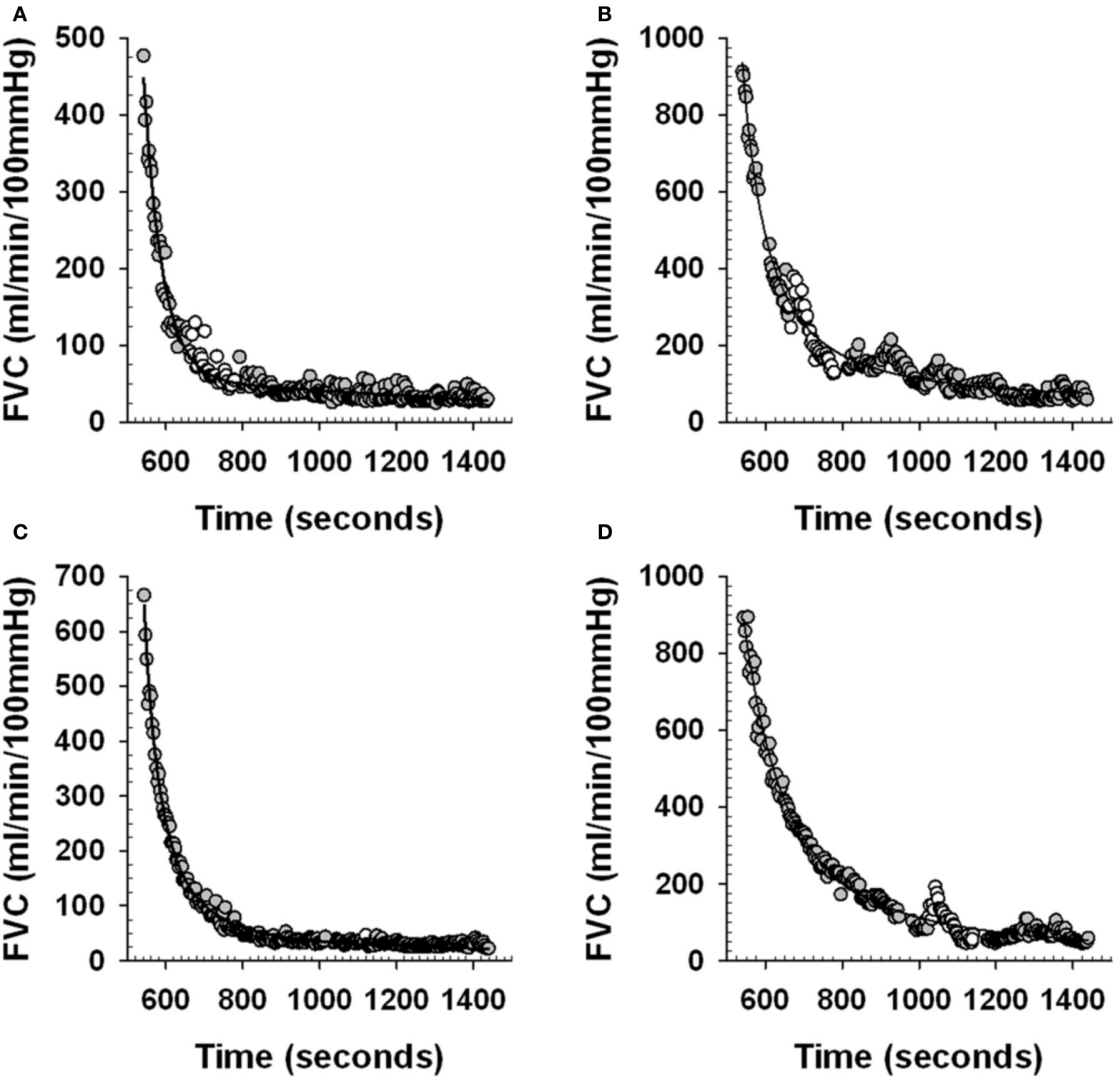
**Examples of curve fit estimation of post-exercise forearm vascular conductance (FVC)**. Panels **(A)** and **(C)** examples of individual subject beat-by-beat post-exercise FVC responses during Control trials (see Figure 1). White circles represent the beat-by-beat FVC data that is removed during the curve fitting to mimic the curve fitting procedure during an actual PE_early_ and PE_late_ CPT trial. Gray circles represent the beat-by-beat FVC data that is curve fit. Solid line is the curve fit. Panels **(B)** and **(D)** examples of individual subject beat-by-beat post-exercise FVC responses during a PE_early_ and PE_late_ trial respectively (see Figure 1). White circles represent the beat-by-beat FVC data that is removed during the curve fitting to allow curve fit estimation of what FVC would have been without the CPT. Gray circles represent the beat-by-beat FVC data that is curve fit. Solid line is the curve fit.

To obtain this FVC_predicted_ the beat-by-beat post-exercise FVC data was averaged into 3 s bins and curve fit using a mono-exponential decay model (Sigmaplot 11.0, Systat Software Inc.), with the CPT data removed. This curve fit then provided an estimate of what FVC would have been during the time at which the last 30 s of the CPT was occurring. We validated this analysis approach as follows. We used the 3 s averaged post-exercise FVC from the control trials (no CPT administered), with a 2 min section of data corresponding to the timing of the CPT in the corresponding post-exercise CPT trials removed. We curve fit these data and compared the curve fit values averaged over the last 30 s of this 2 min period with the 30 s average of the actual FVC (FVC_actual_) (see Figure 2 and Table 2) to ensure that the curve fit approach provided valid estimates of FVC_actual_.

The degree of vasoconstriction due to the CPT in the PE_early_ and PE_late_ CPT trials was then calculated as %reduction in FVC = [(FVC_actual_ – FVC_predicted_)/FVC_predicted_] × 100, where FVC_actual_ was the average of the FVC during the last 30 s of the CPT and FVC_predicted_ was the curve fit estimate of what FVC would have been at that time in the absence of the CPT.

#### CPT catecholamine response: blood sample acquisition

For each experimental trial in which a CPT was performed 6 mL venous blood samples were obtained from the non-exercising (right) arm for 10 s 1–2 min prior to the CPT and immediately after the end of the CPT. After being drawn, blood samples were immediately centrifuged at 1500 Gs for 10 min in a refrigerated centrifuge at 4°C. Plasma was separated from each sample and pipetted into separate 1.5 mL Eppendorf tubes. Plasma samples were immediately stored in a –80°C freezer for catecholamine analysis. Norepinephrine [NE] and Epinephrine [Epi] were analyzed at a later date with a 2-CAT ELISA—Fast Track (Rocky Mountain Diagnostics Inc., Colorado Springs, CO).

### Statistical analysis

The data collected from two subjects were omitted prior to statistical analysis. For one subject this was due to poor data quality (MBV and brachial artery diameter measurements) during data collection. For the other subject, analysis revealed that the CPT did not evoke any forearm vasoconstriction in the Rest CPT trial. Since the CPT was used as a tool to evoke forearm sympathetic vasoconstriction in order to determine if this vasoconstriction was blunted under exercise and post-exercise conditions, failure to evoke vasoconstriction meant that we did not create the required experimental conditions, and justified removal of the subject. As a result, statistical analysis was performed on a total of 8 subjects. All values are reported as means ± SE and significance was set at *P* < 0.05 for all statistical analysis. For all multiple comparison analysis, significant interactions or main effects were analyzed with a Student-Newman-Keuls *post-hoc* test. All data analysis was completed with standard statistical software (SigmaPlot 12.3, Systat Software, Inc.).

#### FVC_predicted_ vs. FVC_actual_

To assess the agreement between the curve fit derived FVC_predicted_ vs. the FVC_actual_ during the PE_early_ and PE_late_ time points in Control trials, we calculated a coefficient of variation between these for each subject within each of the PE_early_ and PE_late_ time points and an intraclass correlation coefficient. In addition, we plotted a linear regression of FVC_predicted_ (x-axis) vs. FVC_actual_ (y-axis) in order to determine if the relationship between the two was on the line of identity, or if a correction factor for conversion of FVC_predicted_ to FVC_actual_ was required.

#### CPT activation repeatability

To confirm that the CPT evoked the same systemic response across experimental days, a Two-Way repeated-measures ANOVA (Time within CPT test, Day of CPT test) was performed on MAP, CO, and TPR. Furthermore, to confirm the CPT evoked the same forearm vasoconstriction between two trials on the same day, as occurred on each experimental day, a Two-Way repeated-measures ANOVA (Time within CPT test, Trial of CPT test) was performed on FVC, and a One-Way repeated-measures ANOVA was performed on the %change in FVC between trials.

#### Catecholamine response

To test the hypothesis that the CPT elevated forearm venous plasma catecholamine concentration similarly in all experimental trials, a Two-Way repeated-measures ANOVA was used to determine the main effect of Trial (Rest, Exercise, PE_early_, and PE_late_) and measurement Time (“Pre-CPT” and “Post-CPT”) and interaction effects of Trial × Time. A One-Way repeated-measures ANOVA was used to compare the TIME (“Pre-CPT” and “Post-CPT”) measurements of NE and Epi from arterialized venous samples during the *Rest* trial to test the hypothesis that CPT did not alter arterial catecholamine concentration.

#### Sympathetic vasoconstriction response to CPT

To test the hypothesis that there was a main effect of trial on the reduction in FVC during the CPT, a One-Way repeated-measures ANOVA was utilized to compare the TRIAL (Rest, Exercise, PE_early_, PE_late_) measurements of %change in FVC from pre- to end-CPT.

## Results

Subject and 15% below foerearm critical force exercise characteristics are presented in Table 1.

**Table 1:** **Subject characteristics**.

	***N* = 8**
Age (years)	21.4 ± 0.8
Height (cm)	179.8 ± 1.3
Weight (kg)	80.3 ± 3.3
Forearm circumference (cm)	25.9 ± 0.5
Forearm volume (mL)	1125.0 ± 49.3
Energy expenditure (METS)	261.0 ± 11.6
Maximal voluntary contraction (kg)	56.4 ± 4.4
Forearm critical force (kg)	29.4 ± 2.4
Exercise at 15% below forearm critical force (kg), (%MVC)	25.0 ± 2.1, 44.9 ± 2.9

Values are means ± S.E. METS—metabolic equivalents. Exercise at 15% below forearm critical force is reported in kg and as a % of maximal voluntary contraction (MVC).

### Curve fitting technique for estimating FVC_predicted_

Figures 2A,C provide an example from one subject of the curve fit approach applied to the control trial post-exercise period that was used to determine the ability for the curve fit to predict where FVC would have been during a normal recovery. The data during the time period where the 2 min CPT would have occurred in the PE_early_ and PE_late_ CPT trials were removed from the post-exercise period of the control trials for that day and then the curve fitting was done, as would be the case during the actual PE CPT trials.

Table 2 presents the agreement between the curve fit predicted FVC and the actual FVC during the last 30 s of this 2 min period. The validity of this curve fit approach for estimating what FVC would have been in the last 30 s of the 2 min CPT period is evidenced by the low coefficient of variation across subjects, the large ICC (0.99 for PE_early_ day, 0.95 for the PE_late_ day) and the lack of difference between the average of the FVC_actual_ and FVC_predicted_.

**Table 2:** **FVC_actual_ and FVC_predicted_ for control trials in PE_early_ and PE_late_ protocols**.

**Subject**	**Control trial PE_early_**			**Control trial PE_late_**		
	**FVC_actual_**	**FVC_predicted_**	**CV (%)**	**FVC_actual_**	**FVC_predicted_**	**CV (%)**
	**(ml/min/100 mmHg)**			**(ml/min/100 mmHg)**		
B	430.6	422.2	1.4	91.7	114.9	15.9
I	131.7	96.5	21.8	102.1	100.8	0.9
K	288.5	249.6	10.2	46.2	49.4	4.6
L	64.7	60.3	5.0	72.0	69.7	2.3
T	103.1	114.2	7.2	67.2	52.7	17.1
V	30.1	36.2	13.1	23.5	22.3	3.6
W	213.6	217.3	1.2	56.0	61.7	6.9
AA	55.5	52.9	3.3	32.5	29.4	7.1
Mean	164.7 ± 48.8	156.2 ± 46.8	7.9 ±2.5	61.4 ± 9.7	62.6 ± 11.4	7.3 ±2.1

Mean ± SE. Control trial PE_early_ and PE_late_, refer to the time during the post-exercise period of the Control trial (**B** in Figure 1) at which the FVC_actual_ and the FVC_predicted_ from the curve fitting procedure (see Figures 2A,C) were obtained. CV—within-subject coefficient of variation.

**Figure 3:**
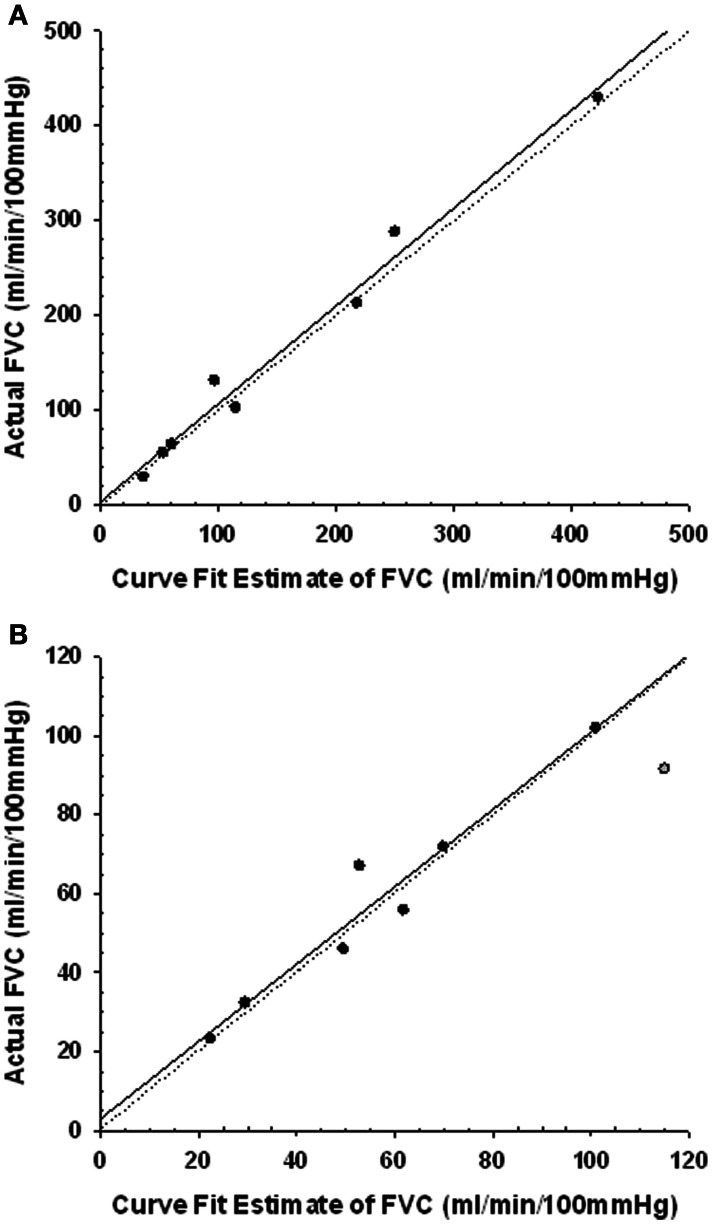
**Regression plots of the curve fit estimated forearm vascular conductance (FVC) and the actual FVC during the last 30 s of the 2 min period in the control post-exercise condition coincident with the timing of the CPT**. Line of identity represented by dotted line. Regression represented by solid line. Panel **(A)** regression for the PE_early_ CPT timing. Panel **(B)** regression for the PE_late_ CPT timing. One subject data point (gray symbol) was excluded from the regression as it had a Cook's *d*-value of 1.6, indicating it had disproportionate impact on the regression parameters.

Figure 3 shows that for the PE_early_ protocol control trials, a regression of the FVC_predicted_ vs. FVC_actual_ values was virtually identical to the line of identity. For the PE_late_ control trials the FVC_predicted_ vs. FVC_actual_ values, although very highly correlated, were represented by a regression that was different from the line of identity. However, on evaluation with a Cook's distance test to assess whether a data point has a disproportionately influential effect on the regression parameters (indicated by a Cook's *d*-value greater than 1), we identified one data point with a Cook's *d*-value of 1.6 (Figure 3). This data point was subsequently omitted from the regression, and the regression then was virtually identical to the line of identity, indicating that our approach provided valid FVC_predicted_.

Figures 2B,D provide an example from one subject of the curve fit approach applied to the PE_early_ and PE_late_ CPT trials. The curve fit was performed on the data with the CPT section of data removed. The comparison between the curve fit and the actual FVC data in the last 30 s of the CPT was used to quantify the magnitude of vasoconstriction.

### Hemodynamic response to the CPT

**Figure 4:**
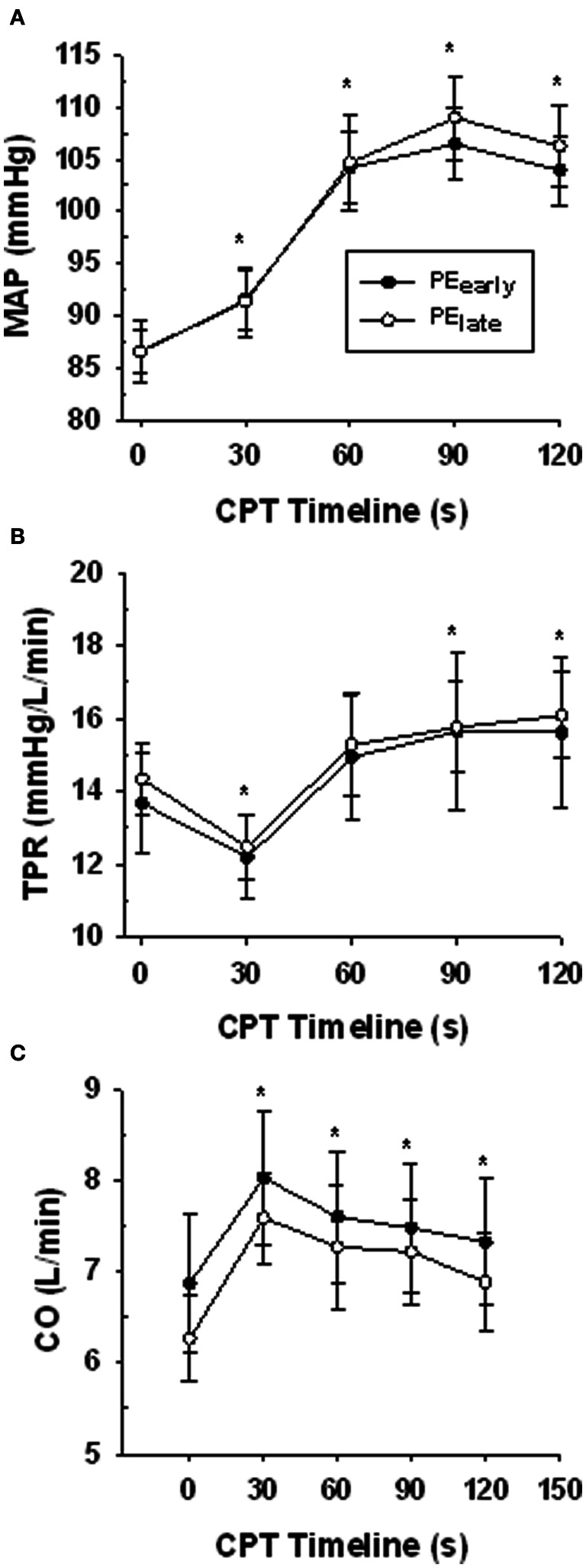
**Central hemodynamic responses over the course of the CPT during the resting trial on the PE_early_ and PE_late_ experimental days**. Panel **(A)** mean arterial pressure (MAP). Panel **(B)** total peripheral resistance (TPR). Panel **(C)** cardiac output (CO). ^*^indicates significantly different from time 0 (baseline) for both PE_early_ and PE_late_ *P* < 0.05.

Table 3 presents the absolute values for HR, MAP, FBF, and FVC for each exercise and post-exercise trial and time point. Figure 4 presents the systemic hemodynamic response to the CPT at rest for both the PE_early_ and PE_late_ days. The CPT resulted in a progressive increase in MAP (main effect of time, *P* < 0.001) but there was no difference between days (main effect of day, *P* = 0.727). The same comparisons for TPR revealed a main effect of time (*P* < 0.001) such that TPR decreased during the first 30 s of CPT compared to pre-CPT and then progressively increased above this nadir over the course of the CPT, such that it was significantly greater than pre-CPT at 90 and 120 s (both *P* = 0.02). This response was consistent between days (main effect of day, *P* = 0.801). Lastly, the same comparisons for CO revealed main effect of time (*P* < 0.001). CO was elevated by 30 s of CPT followed by a steady decline over the rest of the CPT, although it was still significantly elevated compared to pre-CPT at the end of CPT (*P* = 0.02). Again, there was no main effect of day (*P* = 0.595).

**Table 3:** **Absolute hemodynamic responses at all time-points during Trial B–E (see Figure 1)**.

**Time (min)**	**Exercise CPT protocol**		**PE_early_ CPT protocol**		**PE_late_ CPT protocol**	
	**Exercise control trial**	**Exercise CPT trial**	**Exercise control trial**	**PE_early_ CPT trial**	**Exercise control trial**	**PE_late_ CPT trial**
**HEART RATE (bpm)**						
BLN	65.7 ± 4.0	69.9 ± 7.5	64.8 ± 2.2	61.9 ± 1.9	64.7 ± 5.5	66.7 ± 2.6
5	75.8 ± 4.9	85.5 ± 7.4	74.1 ± 3.2	75.6 ± 2.9	69.9 ± 3.3	73.9 ± 3.5
7	77.4 ± 5.8	75.8 ± 5.3	74.4 ± 3.4	79.3 ± 2.6	70.9 ± 3.2	74.1 ± 4.3
9	62.4 ± 5.8	64.9 ± 7.4	62.9 ± 1.7	61.6 ± 2.0	64.7 ± 7.1	63.8 ± 3.2
11	65.3 ± 7.8	66.4 ± 7.8	59.5 ± 2.6	62.8 ± 3.4	59.3 ± 4.0	62.2 ± 4.8
15	65.7 ± 6.5	63.7 ± 7.5	58.4 ± 3.4	59.3 ± 2.7	61.3 ± 4.8	59.7 ± 2.9
17	65.4 ± 5.4	64.8 ± 7.3	59.1 ± 2.1	59.2 ± 2.3	61.6 ± 5.6	63.4 ± 2.4
**MEAN ARTERIAL PRESSURE (mmHg)**						
BLN	92.9 ± 2.4	96.0 ± 2.9	95.0 ± 3.7	95.7 ± 3.0	91.4 ± 2.0	93.3 ± 2.9
5	102.2 ± 3.7	105.6 ± 4.1	108.2 ± 4.3	110.1 ± 3.2	105.1 ± 3.6	105.9 ± 4.0
7	104.6 ± 3.7	118.8 ± 4.2	113.9 ± 5.3	110.6 ± 3.1	106.1 ± 3.6	106.8 ± 3.9
9	91.3 ± 2.2	96.4 ± 3.1	96.5 ± 4.9	96.7 ± 2.2	93.6 ± 2.1	93.7 ± 3.1
11	91.5 ± 2.2	94.2 ± 2.9	95.1 ± 5.1	109.7 ± 3.1	90.5 ± 1.4	95.4 ± 2.3
15	92.2 ± 2.4	94.8 ± 3.1	95.9 ± 4.0	95.4 ± 2.8	91.3 ± 1.6	91.8 ± 2.7
17	92.2 ± 2.6	94.9 ± 3.3	94.6 ± 3.6	95.7 ± 2.2	90.7 ± 1.3	109.1 ± 3.3
**FOREARM BLOOD FLOW (ml/min)**						
BLN	30.7 ± 4.4	34.3 ± 5.3	39.2 ± 5.2	37.5 ± 6.1	32.3 ± 5.0	45.3 ± 12.0
5	376.6 ± 39.7	365.5 ± 28.9	471.0 ± 65.0	464.8 ± 59.3	451.8 ± 67.2	411.9 ± 62.4
7	427.4 ± 48.2	420.5 ± 27.3	520.7 ± 73.7	506.6 ± 71.0	471.9 ± 84.9	469.8 ± 64.6
9	169.4 ± 42.6	153.0 ± 37.9	213.0 ± 52.6	205.7 ± 33.9	222.2 ± 39.0	188.6 ± 37.7
11	112.2 ± 29.4	99.4 ± 24.9	146.2 ± 41.9	100.7 ± 17.5	126.0 ± 29.9	121.5 ± 23.0
15	78.4 ± 17.7	57.4 ± 10.5	87.0 ± 24.6	79.8 ± 11.2	78.0 ± 18.7	65.4 ± 8.6
17	67.0 ± 16.0	51.9 ± 9.8	75.6 ± 17.7	68.4 ± 12.1	59.9 ± 10.2	54.4 ± 6.5
**FOREARM VASCULAR CONDUCTANCE (ml/min/100 mmHg)**						
BLN	34.8 ± 5.3	38.2 ± 4.6	45.8 ± 7.7	41.0 ± 6.2	35.3 ± 6.1	50.1 ± 11.2
5	380.0 ± 36.1	363.8 ± 26.4	459.8 ± 76.1	443.8 ± 65.3	436.2 ± 65.4	405.1 ± 59.9
7	422.4 ± 45.0	370.1 ± 17.8	478.6 ± 77.5	478.9 ± 72.0	452.7 ± 78.7	456.5 ± 65.8
9	178.7 ± 43.8	154.6 ± 37.5	222.5 ± 55.1	217.6 ± 34.9	233.2 ± 38.1	202.6 ± 41.3
11	118.8 ± 30.0	99.7 ± 24.0	148.8 ± 43.4	94.1 ± 16.1	133.0 ± 29.9	121.4 ± 21.9
15	81.5 ± 17.2	55.0 ± 9.0	87.8 ± 24.8	82.9 ± 12.0	79.9 ± 19.1	69.4 ± 10.3
17	71.2 ± 16.0	50.3 ± 8.9	81.9 ± 17.6	73.7 ± 16.4	62.8 ± 9.8	50.8 ± 7.9

All data mean ± SE. BLN—Baseline rest prior to exercise onset. Exercise occurred from end BLN to end 7 min. Post-exercise period began at end of 7 min until 17 min. 5 min—pre-CPT in Exercise CPT Protocol; 7 min—post-CPT in Exercise CPT Protocol; 9 min—pre-CPT in PE_early_ CPT Protocol; 11 min—post-CPT in PE_early_ CPT Protocol; 15 min—pre-CPT in PE_late_ CPT Protocol; 17 min—post-CPT in PE_late_ CPT Protocol. Gray shaded values represent the pre- and post-CPT within the given CPT trial.

### Catecholamine response to the CPT

**Figure 5:**
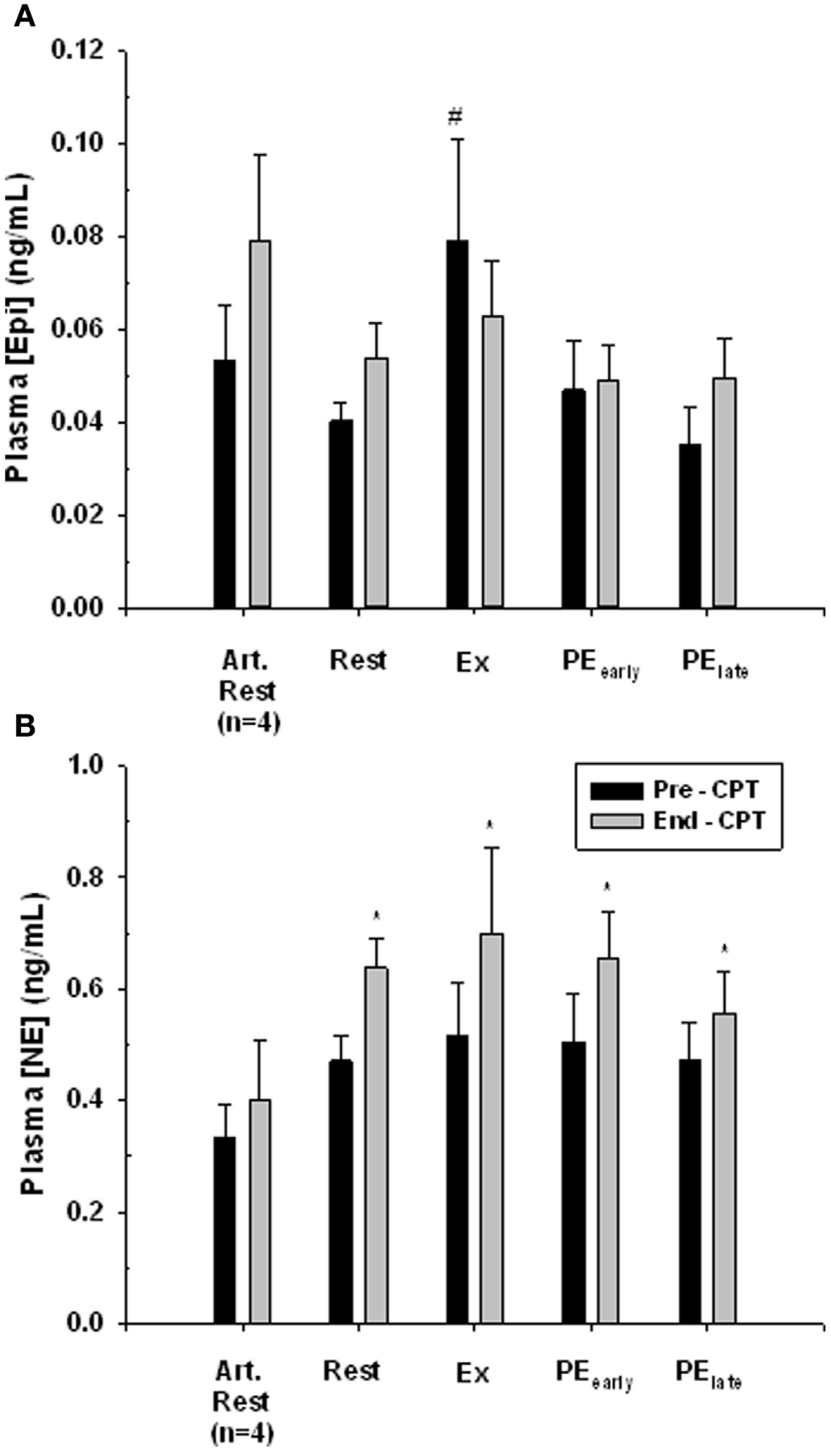
**Catecholamine response to CPT in arterialized venous blood at rest (Art. Rest), and deep forearm venous blood at rest, exercise (Ex) early post-exercise (PE_early_) and late post-exercise (PE_late_)**. Panel **(A)** plasma epinephrine concentration. ^#^ indicates significantly different from all other Pre-CPT forearm deep venous values, *P* < 0.05. Panel **(B)** plasma norepinephrine concentration. ^*^indicates End-CPT significantly different from corresponding Pre-CPT, *P* < 0.05.

The response of venous plasma [NE] and [Epi] concentration to the administered CPT is portrayed in Figure 5. The CPT resulted in a significant rise in forearm deep venous plasma [NE] for all trials (main effect Pre- to End-CPT *P* = 0.001) and there was no interaction effect (*P* = 0.777). One-Way repeated-measures ANOVA comparison of the increase in venous plasma [NE] between rest, exercise, PE_early_, and PE_late_ indicated no difference (*P* = 0.500). There was no significant increase (*P* = 0.32) in the arterialized plasma [NE] response to the CPT assessed during the Rest trial. The arterialized plasma [Epi] concentration did not change with CPT (*P* = 0.152), nor did the forearm deep venous plasma [Epi] concentrations (Main effect of Pre- to End-CPT *P* = 0.595). However, there was an interaction between trial and Pre- to End-CPT such that the Pre-CPT during Exercise was greater than Rest (*P* = 0.004), PE_early_ (0.007), and PE_late_ (*P* = 0.002).

### Forearm vasoconstrictor response to the CPT

**Figure 6:**
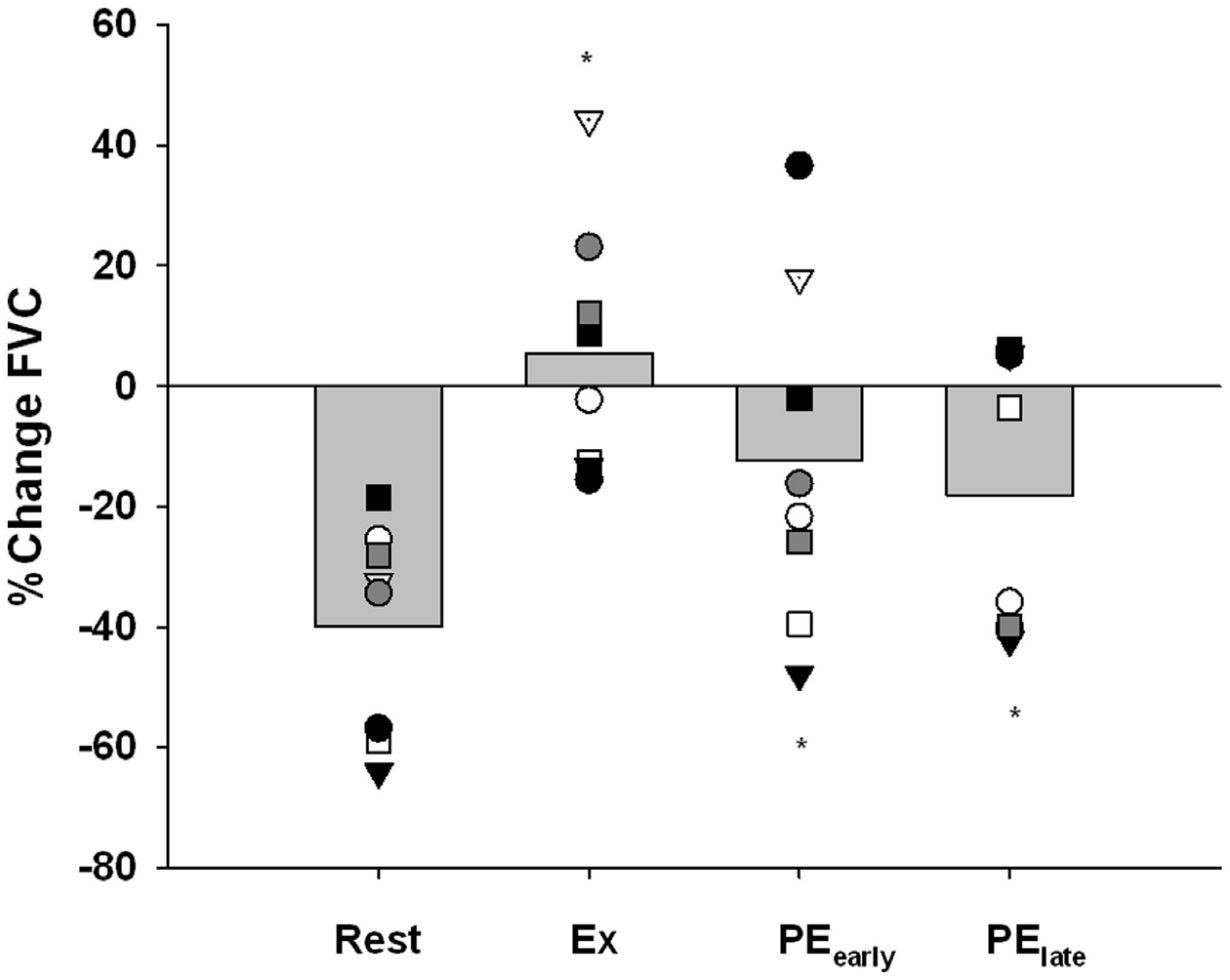
**Percent change in forearm vascular conductance (FVC) in response to CPT**. The response of each subject is identified with a unique symbol. ^*^indicates significantly different from Rest, *P* < 0.05.

Figure 6 presents the vasoconstrictor response to CPT as %change FVC. All subjects demonstrated a severely blunted to completely abolished vasoconstriction when CPT was administered during exercise. In contrast, 6 of 8 subjects in PE_early_ and 5 of 8 subjects in PE_late_ still demonstrated a blunted vasoconstriction. At the group level, a One-Way repeated-measures ANOVA and subsequent *post-hoc* analysis identified that all trials were significantly different from rest (Exercise: *P* < 0.001, PE_early_: *P* = 0.022, PE_late_: *P* = 0.032).

While *P*-values for the comparison of PE_early_ vs. Exercise and PE_late_ vs. Exercise were 0.073 and 0.052, respectively and therefore did not reach the a priori 0.05 threshold for accepting that the differences are not likely to be due to chance, the inadequate power of the study to detect these differences must be considered when interpreting the data.

### Time control experiment forearm vasoconstrictor response to CPT

**Figure 7:**
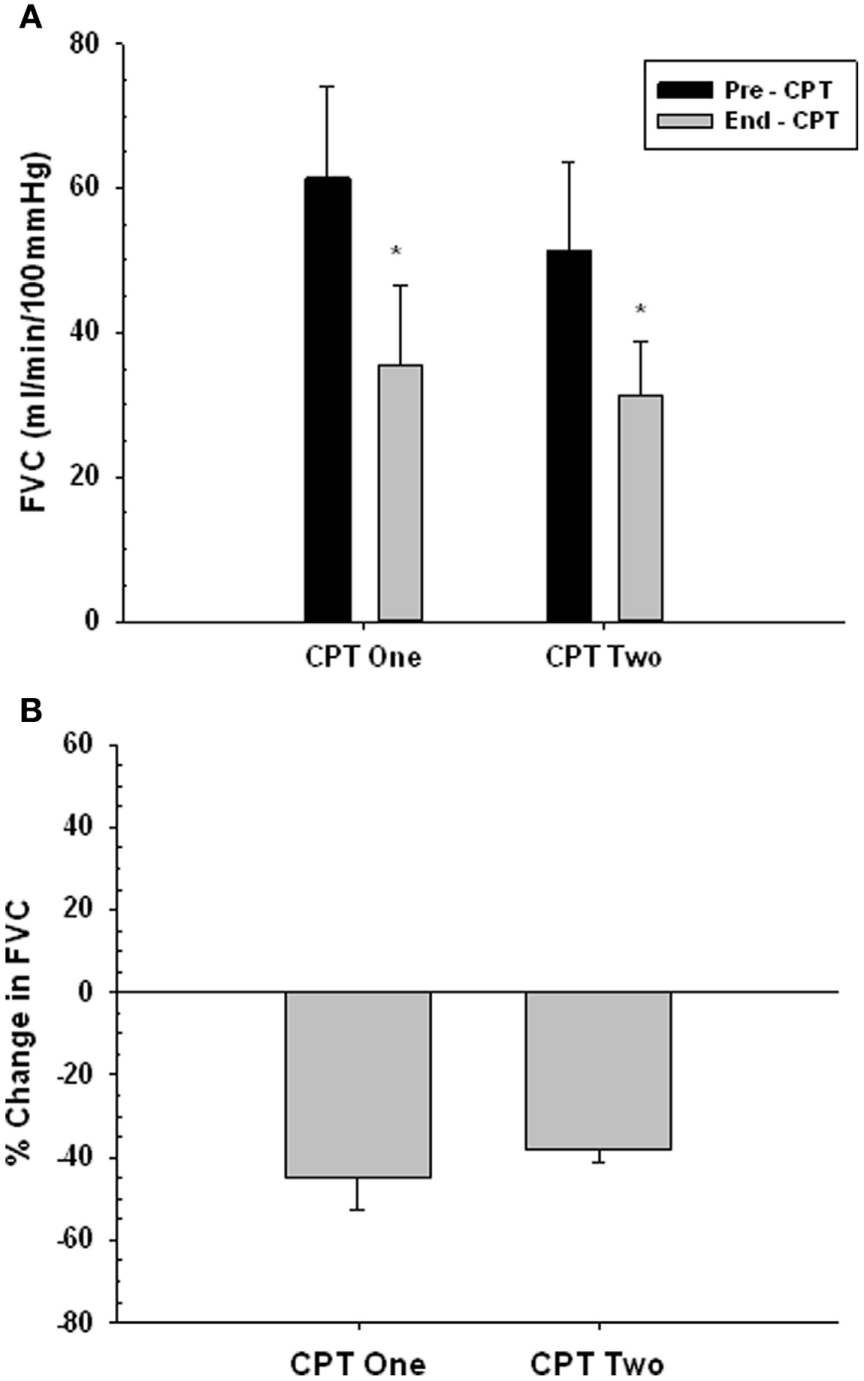
**Forearm vasoconstrictor response to CPT during repeated trials at rest**. CPT 1—first CPT test. CPT 2—second CPT test. Panel **(A)** forearm vascular conductance (FVC) before (Pre-CPT) and at the end of (End-CPT) a CPT. Panel **(B)** The difference in Pre- vs. End-CPT FVC expressed as %change. ^*^Main effect, significantly different from Pre-CPT, *P* = 0.002.

Figures 7A,B present the vasoconstrictor response to CPT at rest for two trials, used to confirm that the vasoconstrictor response to a second CPT trial on a given day was maintained. There was no main effect of trial (i.e., Pre- and End-CPT FVC was not different between trials, *P* = 0.271), but there was a main effect of time (i.e., End-CPT FVC was less than Pre-CPT, *P* = 0.002). Furthermore, the %reduction in FVC in CPT 1 vs. CPT 2 was not different (*P* = 0.405).

## Discussion

This study was the first to test the hypothesis that functional sympatholysis persists in human skeletal muscle in the immediate (10 min) post-exercise period. Functional sympatholysis is a blunting of sympathetic vasoconstriction due to mechanisms activated within skeletal muscle as a consequence of muscle contractions. The nature of these mechanisms means that they would not disappear immediately with cessation of muscle contractions as their presence is not contingent on the mechanical or muscle activation aspects of muscle contraction which disappear immediately at end exercise. We have therefore termed the blunted sympathetic vasoconstriction in the first 10 min of exercise as a persistence of functional sympatholysis rather than referring to it as a separate sympatholytic phenomenon.

The primary findings of this study were as follows. First, assessment of the central hemodynamic response to CPT in the Rest trial on each of the two post-exercise testing days (PE_early_ and PE_late_) confirmed the repeatability of the CPT effect between days. Namely, an initial CO-mediated pressor response transitioned into continued increases in MAP resulting from increasing TPR while CO was declining. When taken together with observations of a robust forearm vasoconstriction at rest, and elevated forearm venous plasma [NE] responses to the CPT in all trials, it is clear that the CPT evoked sympathetic forearm vasoconstriction. Second, in a separate time control experiment we confirmed that the forearm vasoconstrictor response to CPT does not diminish with a repeated CPT test. Third, robust functional sympatholysis was observed during exercise, confirming the activation of these mechanisms. Finally, and most importantly, the reduction in FVC in response to the CPT at 4 min (PE_early_) and 10 min (PE_late_) post-exercise was blunted compared to Rest. These observations support the concept of a lingering functional sympatholysis effect for as long as 10 min following human skeletal muscle exercise at 15% below critical force.

### The use of the CPT to evoke increased sympathetic vasoconstriction

In this investigation, the CPT was a reliable methodological approach for elevating forearm sympathetic vasoconstriction and therefore allowing the assessment of functional sympatholysis. The administration of the CPT resulted in repeatable elevations of forearm deep venous plasma [NE] in all experimental conditions and a robust forearm vasoconstriction at rest. Previous evidence has also linked the use of the CPT with elevations in venous plasma [NE]. Winer and Carter (1977) observed significant increases in plasma [NE] during hand immersion in ice water that was linked to elevations in MAP and HR. Stratton et al. (1983) also observed significant elevations in [NE] after 6 min of CPT administration as compared to rest. These observations confirm the use of the CPT for elevating forearm vasoconstrictor activity.

### Functional sympatholysis during 15% below fCF exercise

In the present study, the administration of the CPT during Rest resulted in a substantial reduction in FVC (−39.9 ± 6.1%), indicating that sympathetically-mediated vasoconstriction is robust at the skeletal muscle during resting periods. The mean percent change in FVC due to the CPT during Exercise indicated that the vasoconstrictor response appeared to be abolished at the skeletal muscle during exercise at 15% below fCF.

These primary findings are in accordance with a wealth of literature demonstrating the presence of functional sympatholysis in exercising muscle. This includes studies that specifically used CPT to elevate sympathetic vasoconstriction. For example, Wray et al. (2007) also utilized the CPT in their investigations into sympathetic vasoconstriction during exercise in cyclists and sedentary humans. Similar to the findings reported in this investigation, they demonstrated a significant reduction in FVC during CPT administration at rest (−38 ± 6%) which appeared abolished during 50% maximal voluntary contraction handgrip exercise (13 ± 11%). Differences between our study forearm exercise and theirs were the use of isometric rather than dynamic exercise contractions, a contraction force on average of ~44% of MVC, and a 1 s contraction to 2 s relaxation duty cycle compared to their 1:1 duty cycle. The latter two characteristics indicate the exercise intensity in our study was therefore considerably less. In fact the moderate increase in arterial blood pressure from rest to exercise (see Table 3) and the lack of increase in forearm venous effluent NE (see Figure 5) is consistent with a sustainable exercise intensity we have used previously to investigate functional sympatholysis (Tschakovsky et al., 2002).

Despite blunting of exercising muscle vasoconstriction, the carotid baroreflex recruitment of exercising muscle sympathetic restraint for the purpose of blood pressure regulation can occur during exercise. This is evidenced by blood pressure elevation and exercising leg vasoconstriction in response to simulated carotid hypotension via neck compression (Keller et al., 2004). However, it must be noted that to date no studies have determined whether blood pressure regulation in response to an actual hypotensive disturbance during exercise is adequately preserved. In other words, whether functional sympatholysis can interfere with blood pressure regulation in the face of sudden hypotension. Furthermore, impairment in blood pressure regulation in response to a hypotensive disturbance might be even greater post-exercise, where the muscle pump is not supporting central venous filling pressure.

### Functional sympatholysis post-exercise

We developed a new analysis approach to detect blunting of sympathetic vasoconstriction during a non-steady state period immediately following exercise. This technique has very good predictive validity (curve fit estimation of FVC during the post-exercise period closely matched actual FVC), and provides a new research tool for understanding sympathetic vasoconstriction in muscle post-exercise.

The most important finding of the current study was that sympathetic vasoconstriction remained significantly blunted in the human forearm at both 4 and 10 min post-exercise. The possibility also existed for us to be able to assess whether the potency of sympatholysis diminished over this time period. We would hypothesize that over time, sympatholytic molecules like ATP, NO, and H^+^ would diminish, and if they were responsible for sympatholysis in the acute post-exercise period, the potency of sympatholysis would diminish as well. *Post-hoc* analysis identified a *P* = 0.073 for PE_early_ vs. Exercise, and *P* = 0.052 for PE_late_ vs. Exercise. Strictly speaking, failure to achieve *P* < 0.05 that is set a priori requires we interpret the findings to suggest there was not a diminished functional sympatholysis as much as 10 min post-exercise. However, given the sample size and variability in the data, it is quite likely that this interpretation would represent an incorrect failure to reject the null hypothesis.

An additional important consideration in this regard is the possibility of individual differences in the time course of sympatholytic molecule, and therefore sympatholysis, disappearance. By 10 min, three of eight subjects appeared to have restored sympathetic vasoconstriction with CPT, while five maintained robust sympatholysis. Group level data interpretation may become problematic when there are apparent individual differences in the time course of functional sympatholysis decay. Follow-up investigations with a larger subject pool with repeated measures within each individual at multiple post-exercise assessment time points are necessary to better determine the exact timeline of functional sympatholysis decay post-exercise and its variation between individuals.

Our findings in humans are consistent with those of Howard and DiCarlo (1992) in rabbits, who observed a blunting of vasoconstriction in response to phenylephrine infusion 10 min post-exercise compared to rest. This finding was later replicated by the same group (Patil et al., 1993) who utilized phenylephrine infusion and bouts of treadmill exercise to examine alpha-adrenergic receptor-mediated vasoconstrictor response post-exercise in Sprague–Dawley rats. In the exercise trial, rats were infused with phenylephrine then ran on a treadmill at 12–18 m/min at a 10–18% incline until exhaustion (mean = 45 min). These investigators observed a blunted vasoconstrictor response to phenylephrine infusion at 6 min post-exercise. In addition, they examined the effect of nitric oxide synthase (NOS)blockade and found that both at rest and after exercise, this resulted in more robust vasoconstriction in response to phenylephrine.

Whether nitric oxide is responsible for post-exercise sympatholysis remains to be determined. Previous evidence suggests a role for NO in the regulation of vasomotor tone following skeletal muscle exercise. Radegran and Saltin (1999) investigated the impact of NOS inhibition (via L-NMMA infusion) on the femoral artery blood flow response following submaximal one-legged, dynamic knee-extensor exercise. Ten minutes post-exercise, these investigators observed a significant reduction of 66 ± 5% in femoral artery blood flow during exercise + L-NMMA infusion as compared to exercise alone. Shoemaker et al. (1997) found that brachial artery infusions of L-NMMA also reduced post-exercise forearm blood flow following handgrip exercise.

#### Advantages and potential limitations

A critical characteristic of the present study was the inclusion of time control experiments which confirm the reproducibility of the CPT vasoconstrictor response with repeated trials. Since the resting CPT trial was always performed before the exercise or post-exercise CPT trials, the possibility existed that blunted sympatho-excitation response to a second CPT could explain the reduced vasoconstriction compared to rest. The control experiments confirmed that this was not the case. We observed no difference in the vasoconstrictor effect of CPT across two trials that were separated by the amount of time that occurred between rest CPT and the experimental post-exercise CPT's.

Another strength of the current study is that participants were screened prior to recruitment in order to ensure that only participants that would demonstrate sympathetic vasoconstriction as the major component of their CPT response were included in the study. Remembering that the CPT was merely a tool to evoke sympatho-excitation and forearm vasoconstriction, it was important to ensure that this tool did indeed evoke what it was intended to evoke. A lack of participant screening prior to investigation when utilizing the CPT could easily lead to confounded conclusions due to the non-homogeneous response to the CPT within a population (Ifuku et al., 2007; Moriyama and Ifuku, 2007, 2010). Furthermore, we accounted for normal changes in post-exercise blood flow by validation of a curve fit prediction technique that provided good estimates of what the normal FVC would have been during the CPT test.

A potential limitation of the study is the inability to conclusively confirm that underlying MSNA was the same across experimental conditions. In the current study, we wished to determine whether post-exercise functional sympatholysis persisted. For this reason, we chose a work rate that would maximize potential sympatholytic mechanisms during exercise so that detection of their persistence post-exercise would be enhanced. Additionally, in order to assess the effect of additional sympatho-excitation during exercise, it was necessary for the exercise intensity to result in a steady state. Given the problems with %MVC representing a given relative metabolic intensity, (Kent-Braun et al., 1993; Saugen et al., 1997) we identified an exercise intensity of 15% below fCF, which maximizes the exercise intensity while still safely ensuring the achievement of metabolic steady state in exercise. An important consideration when interpreting the responses to an imposed sympatho-excitation is the potential confound of underlying MSNA levels. In other words, is comparison of the effect of sympatho-excitation (1) at rest vs. during exercise at 15% below fCF confounded by different levels of underlying MSNA, and (2) at rest vs. post-exercise confounded by withdrawal of exercise-evoked MSNA during recovery?

In answer to the first issue: measurement of forearm venous effluent [NE] (see Figure 5B) confirmed that there was no increase in response to exercise. Yet there was a robust and virtually identical increase in forearm venous effluent [NE] in response to CPT at rest and during exercise. Finally, the exercise intensity in this present study resulted in similar FVC and MAP increases from rest to exercise as in our previously published work which established the exercise intensity dependence of functional sympatholysis in exercising human forearm (Tschakovsky et al., 2002). Taken together, these findings support our interpretation of robust functional sympatholysis during exercise at 15% below fCF in the present study.

In answer to the second issue: it might be argued that post-exercise withdrawal of any exercise-induced elevations in MSNA could serve as a “passive” dilatory signal, explaining the reduced vasoconstriction in response to CPT post-exercise. For this to be the case, the post-exercise withdrawal of MSNA would have to be occurring concomitant with the CPT stimulation of MSNA, resulting in a net blunting of the increase in MSNA. Arguing against this is the fact that already by the time the early post-exercise CPT is initiated, HR and MAP have returned to resting baseline levels, indicating a rapid return to baseline of systemic sympatho-excitation. Furthermore, there is no difference in the end-CPT forearm venous effluent [NE] response in the rest vs. the two post-exercise CPTs. For all *in vivo* studies of functional sympatholysis the basal MSNA upon which a sympatho-excitatory stimulus is superimposed is not known. Whether different basal MSNA affects the response to CPT MSNA elevation is also not known. Therefore, as in all such studies, the possibility that a difference in sympatho-excitation evoked vasoconstriction between conditions is due to underlying MSNA differences cannot be conclusively ruled out. In this regard the present study is not different from previous work in this area.

Finally, another potential limitation of the present study is the *n* = 8 due to the loss of two subjects, which decreases statistical power. In the case of the difference between resting and post-exercise %reduction in FVC, statistical significance was reached. However, it is possible that for the catecholamine analysis, specifically the epinephrine response, differences were not detected due to lack of statistical power. It is also possible that a failure to reject the null hypothesis in comparing functional sympatholysis potency between exercise and post-exercise conditions is due to a lack of statistical power.

#### Implications

This is the first study to confirm lingering functional sympatholysis in human forearm muscle post-exercise. The implications of these findings must be considered within the context of the subject selection criteria. These were young, healthy males who demonstrated a vasoconstrictor response to CPT sympatho-excitation. The data cannot be generalized to persons outside of these criteria. Since sympatholysis during exercise occurs in both upper (Tschakovsky et al., 2002; Wray et al., 2007) and lower limb (Wray et al., 2007) exercise in humans, it is likely that sympatholysis would linger post-lower limb exercise as well. The persistence of functional sympatholysis following exercise with large muscle mass holds significance for athletes and the modern day exercise-enthusiast. Following an endurance exercise task, an athlete may experience light-headedness. This is in part due to the relaxation of the muscle pump, which leads to a decline in venous return and an acute reduction in filling pressure of the heart. Combined with prolonged muscle vasodilation post-exercise, this could ultimately result in syncope. Prevention of syncope via baroreflex-mediated sympathetic vasoconstriction at a time when CO increase may be limited would clearly be difficult due to the prolonged blunting of vasoconstriction at the previously exercised skeletal muscle.

Factors which impair blood pressure regulation in the face of a hypotensive disturbance have serious implications for fighter pilots (Scott et al., 2007). Because they complete full body contractions in order to avoid gravity-induced loss of consciousness (GLOC), the persistence of functional sympatholysis in the large muscle mass used for this could be problematic during subsequent mild +Gz maneuvers following these muscular efforts where pilots may not engage in countermeasure contraction. In this context, further research into the duration, magnitude, and individual differences in post-exercise functional sympatholysis is warranted. This would include determining whether blood pressure regulation in response to +Gz is compromised post-exercise.

## Conclusions

In conclusion, this study has demonstrated for the first time in humans the existence of a lingering functional sympatholysis in the first 10 min following a bout of forearm exercise. This may have important implications for blood pressure regulation in the face of a hypotensive challenge. In this context, differences between individuals in the duration that functional sympatholysis is maintained post-exercise may also need to be considered.

### Conflict of interest statement

The authors declare that the research was conducted in the absence of any commercial or financial relationships that could be construed as a potential conflict of interest.
